# Microbiota characterization throughout the digestive tract of horses fed a high-fiber vs. a high-starch diet

**DOI:** 10.3389/fvets.2024.1386135

**Published:** 2024-05-14

**Authors:** Federica Raspa, Stefania Chessa, Domenico Bergero, Paola Sacchi, Ilario Ferrocino, Luca Cocolin, Maria Rita Corvaglia, Riccardo Moretti, Damiano Cavallini, Emanuela Valle

**Affiliations:** ^1^Department of Veterinary Sciences, University of Turin, Grugliasco, Italy; ^2^Department of Agricultural, Forestry and Food Science, University of Turin, Grugliasco, Italy; ^3^Department of Veterinary Sciences, University of Bologna, Bologna, Italy

**Keywords:** horse, 16S rRNA, microbiota, nutrition, welfare

## Abstract

**Introduction:**

Diet is one of the main factors influencing the intestinal microbiota in horses, yet a systematic characterization of the microbiota along the length of the digestive tract in clinically healthy horses, homogenous for age and breed and receiving a specific diet is lacking.

**Methods:**

The study used 16S rRNA amplicon sequencing to characterize the microbiota of the intestinal tracts of 19 healthy Bardigiano horses of 14.3  ±  0.7  months of age fed one of two diets. Nine horses received a high-starch diet (HS), and ten horses received a high-fiber diet (HF). After 129  days, the horses were slaughtered, and samples were collected from the different intestinal tract compartments.

**Results and discussion:**

The microbiota alpha diversity indices were lower in the caecum, pelvic flexure and right dorsal colon of the horses fed the HS diet (False Discovery Rate, FDR  <  0.05). The values of beta diversity indicated significant compositional differences between the studied intestinal tract compartments according to the diet received (FDR  <  0.05). At the lower taxonomic level (genus or family), the HS diet was associated with a higher relative frequency of *Enterobacteriaceae* within the small intestine (jejunum and duodenum) (FDR  <  0.05). Within the hindgut (caecum and sternal flexure), the HS diet was associated with lower relative frequencies (i.e., a smaller core community) of bacteria belonging to *Fibrobacteraceae* and *Prevotellaceae* (FDR  <  0.05). Moreover, horses fed the HS diet displayed a higher relative abundance of *Streptococcus* in the caecum (FDR  <  0.05) and *Fusobacterium* in the sternal flexure (FDR  <  0.05), both of which are pathogenic bacteria responsible for inflammation diseases. Samples collected from the pelvic flexure and rectum of horses fed the HS diet showed significantly higher relative frequencies of *Succinivibrionaceae* (FDR  <  0.05) – amylolytic bacteria associated with acidosis. The relative frequencies of the *Lachnospiraceae* and *Ruminococcaceae* were lower in the feces collected from the rectum of horses receiving the HS diet vs. HF diet, indicating smaller core communities of these bacteria (FDR  <  0.05). Fibrous diets should be promoted to prevent dysbiosis of the microbiota associated with high-starch diet.

## 1 Introduction

A better understanding of the etiology of digestive disorders is crucial for safeguarding horse welfare and digestive health (1). Several factors related both to intrinsic to the individual and to management conditions can predispose the horse to the onset of digestive disorders (2). One of the main risk factors of digestive disorders is being fed a diet high in starch and low in fiber (3). In fact, the horse’s capacity to digest starch is highly limited due to their low production of pancreatic α-amylase (4, 5). Accordingly, several authors agree that starch consumption should be limited to not more than 1 gram of starch/kg bodyweight (BW)/meal (6–9). Despite this recommendation, horses are commonly fed diets characterized by much higher amounts of starch (10–12). Such a feeding practice results in an impairment of the gut health, causing hindgut acidosis, dysbiosis and colic (13). Systemic consequences are also possible, such as laminitis, weight loss and poor performance (1, 14, 15). The presence of indigested starch in the hindgut can trigger microbial population changes during the fermentation processes (5, 16), yet little research effort has been directed at characterizing the horse microbiota in the different compartments of the intestinal tract according to diet with respect to other animal species (17–19). De Fombelle et al. (20) applied a culture-based methodology to samples obtained from the jejunum, ileum, caecum, right ventral colon and left dorsal colon; this approach thus limited the output to cultivable species only (21). Dougal et al. (22) investigated microbial population communities using a PCR-based method on samples from the caecum, right dorsal colon and rectum of eight equines euthanized for reasons not related to metabolic or intestinal disturbances. However, neither culture-dependent nor PCR-based methodologies permit the full richness and diversity of the bacterial populations resident in the gut to be characterized (23). To the best of our knowledge, Dougal et al. (24) used an amplicon sequencing approach to identify the core bacterial community across seven different regions of the large intestine of ten equines. Moreover, the study carried out by Costa et al. (18) applied a sequencing approach to evaluate the microbiota of the horse in the different compartments of the intestinal tract. However, this latter study only involved eleven horses belonging to different breeds, characterized by a wide age range (2 to 30 years) and which were euthanized for pathologies (unrelated to gastrointestinal diseases). The goal of the present study was to deepen our understanding of the microbial differences in different intestinal tract compartments in healthy horses, homogenous for breed and age, according to the diet being fed, namely a high-fiber diet (HF) vs. a high-starch diet (HS). We hypothesized that that differences in the gut microbiota in the distinct intestinal tract compartments would be observed between the two dietary groups. This study aims to further our understanding of the microbial changes associated with a HS diet in the intestinal environment.

## 2 Materials and methods

The present study forms part of a larger research project investigating the effects of a high-fiber diet (HF) vs. a high-starch diet (HS). The same horses were also investigated for other parameters of gut health as gut histomorphometry (25) and fermentation end-products (16); behavior (26) and growing performances (15).

The study followed the guidelines of the current European Directive (2010/63/EU) on the care and protection of animals, and it was approved by the Ethics Committee of the Department of Veterinary Sciences of the University of Turin (Italy) (Prot. N. 2,202/2019).

### 2.1 Animals and feeding management

The study was performed in Piedmont, Northwest Italy between June and October 2019. Nineteen healthy Bardigiano horses of 14.3 ± 0.7 months of age (mean ± standard deviation, SD) were enrolled in the study. Upon their arrival at the farm, all horses were dewormed using an oral gel preparation (1.29 g/100 kg BW, Eqvalan Duo, Merial Animal Health). The horses were first acclimatized to the farm environment in an outdoor paddock area for two weeks before being randomly assigned to two indoor group pens for the following 129 days until slaughter. The group pens were situated inside a barn with two open sides and no access to any outdoor paddock area. Pens were enclosed by horizontal metal rail bars, which also delimited the pens at the feed bunk. The horses fed the HF diet were 7 fillies and 3 colts with a mean ± standard deviation (SD) initial bodyweight (BW) of 221.10 ± 5.00 kg; the horses fed the HS diet were 5 fillies and 4 colts with a mean ± SD initial BW of 217.56 ± 9.28 kg. The horses belonging to the HF and HS groups received the same first-cut meadow hay, but different complementary feeds. Horses were individually fed the complementary feeds that were gradually increased to reach the final amount during the last 72 days of the fattening period (15). Briefly, for the HS, the amount (as fed) of the starch-rich pelleted feed was gradually increased as follow: 3 kg/animal/day for the first 13 days, followed by 4.5 kg/animal/day for the subsequent 6 days, 5 kg/animal day for further 36 days, and 8 kg/animal/day during the last 72 days of the fattening period. Those quantities were fed according to the usual feeding practice adopted by the farmer. For the HF, the amount (as fed) of the fiber-rich pelleted feed was gradually increased as follow: 1 kg/animal/day for 7 days, 2 kg/animal/day for 9 days, 2.5 kg/animal/day for 25 days, 3 kg/animal/day for 9 days, and finally 3.5 kg/animal/day until the end of the fattening period (72 days). The HF diet was planned by a veterinary nutritionist according to the nutritional requirements of the French “Institute National de la Research Agronomique” (INRA) (27).

During the last 72 days of the fattening period, each horse belonging to the HS received 8 kg/horse/day of the starch-rich pelleted feed containing 5.70 g (as fed) of starch per kg BW/meal, whereas each horse belonging to the HF group received 3.5 kg/horse/day of the fiber-rich pelleted feed containing 0.97 g (as fed) of starch per kg BW/meal. The complementary pelleted feeds were individually supplied to the horses twice a day (7 am and 6 pm). Hay was provided at the same time, and the hay consumption was 8 kg/horse/day for HF and 6 kg/horse/day for HS. The ingredients and the chemical composition of hay and complementary feeds are shown in Table 1. The daily nutritional composition of the diets (hay plus feed) are shown in Table 2.

**Table 1:** Chemical composition of hay and complementary pelleted feeds.

	Hay	Fiber-rich pelleted feed^1^	Starch-rich pelleted feed^2^
Dry matter, g/kg	898	906	899
Crude protein, g/kg	73.7	218.2	158.1
Ether extract, g/kg	11.5	55.8	41.0
Crude fiber, g/kg	33.4	127.3	49.4
Ash, g/kg	69.4	118.9	92.3
Starch, g/kg	not analyzed	210.9	550.6
Net Energy, MJ/kg	4.0	7.9	10.3

Values are expressed on dry matter basis. ^1^Ingredients, % of inclusion as fed: soybean hulls (12%), soybean meal (12%), wheat bran (10%), beet pulp (10%), rice flower (10%), wheat flour (8%), extruded soybean (8%), alfalfa hay (5%), carobs (3%), molasses (3%), sunflower meal (4.5%), linseed (3.5%), calcium carbonate (4%), calcium phosphate, sodium chloride (3%), (3%), vitamin and mineral mix (1%, Vit A = 25,000 UI, Vit D3 = 500 UI, Vit E = 270 mg, Vit B1 = 18 mg, Vit B2 = 8 mg, Vit B6 = 13 mg, Vit C = 85 mg, Vit K = 30 mg, Niacinamide = 21 mg, Folic acid = 4 mg, Fe = 22 mg, Cu = 22 mg, Zn = 62 mg, Mg = 35 mg, I = 5 mg, Co = 2 mg, Se = 0.4 mg). ^2^Ingredients, % of inclusion as fed: cracked corn grain (57%), wheat bran (9%), soybean meal (8%), beet pulp (4%), corn gluten meal (4%), sunflower meal (4%), wheat flour (4%), molasses (3%), calcium carbonate (2.5%), sodium chloride (2%), sodium bicarbonate (2%), vitamin and mineral mix (0.5%, contained per kg of vitamin supplement: Vit A = 17,000 IU, Vit D3 = 2,500 IU, Vit E = 35 mg, Vit B1 = 1 mg, Cu = 10 mg, Fe = 13 mg, Mg = 35 mg, Zn = 87 mg).

**Table 2:** Nutritional composition of the overall daily diets (hay plus feed) during the fattening period (72 days).

	High fiber diet	High starch diet
Hay, kg/horse/day	8.9	6.7
Feed, kg/horse/day	3.9	8.9
Crude protein, g/horse/day	1499.7	1899.1
Ether extract, g/horse/day	317.9	441.9
Crude fiber, g/horse/day	789.3	662.7
Ash, g/horse/day	1077.7	1285.1
Starch, g/horse/day	814.8	4899.8
Starch, g/kg bodyweight/meal	1.2	7.0
Net Energy, MJ/horse/day	66.4	118.8

Values are expressed on dry matter basis.

### 2.2 Sample collection

Animals were slaughtered 129 days after their arrival at the farm, at the end of the fattening period. The slaughtering procedures were performed according to European Union regulations (EU 2009/853 and EU 627/2019) under the supervision of official veterinarians.

**Figure 1:**
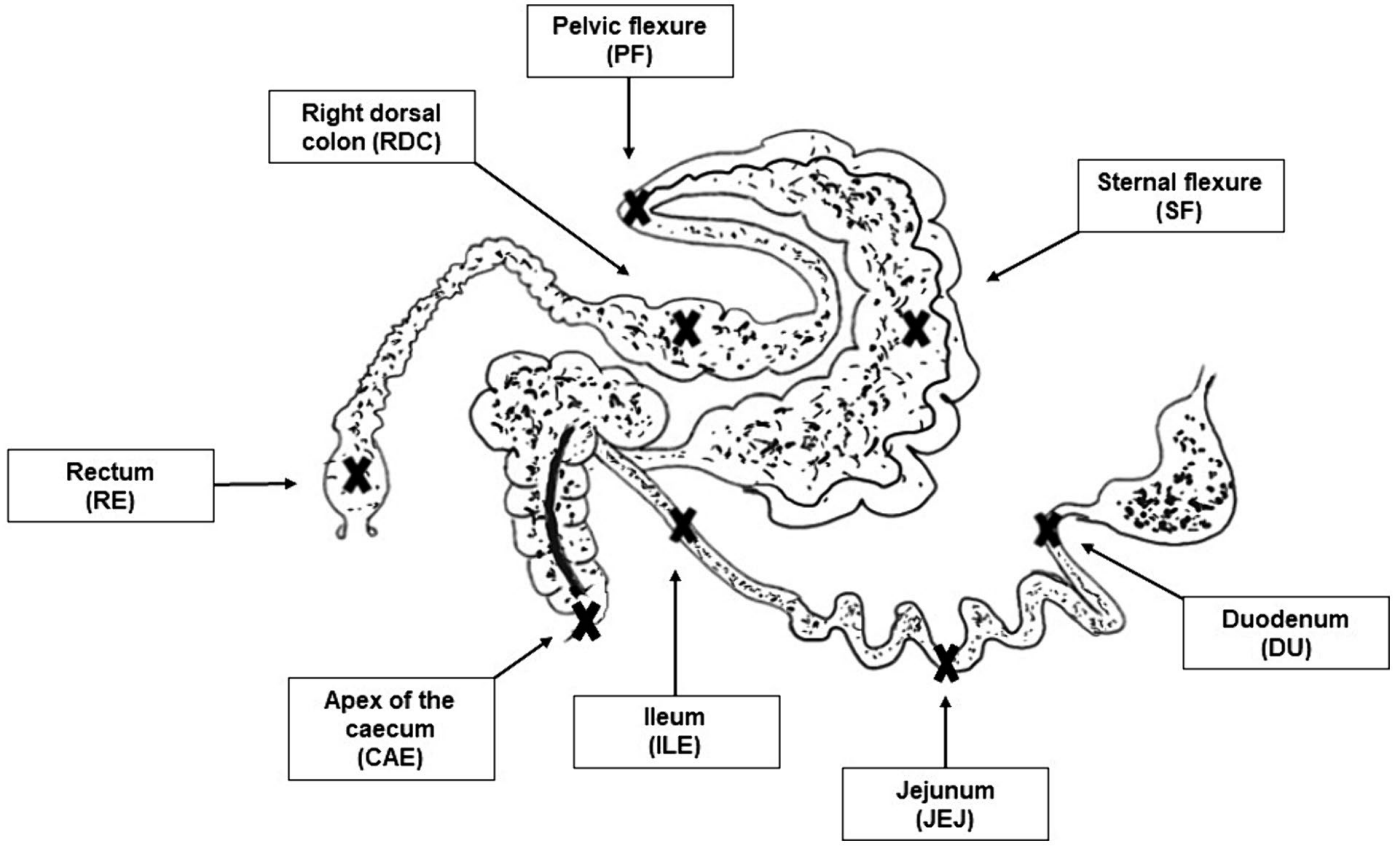
Picture of the sampling sites. Crosses indicate the sampling sites for the selected intestinal tract compartments: duodenum (DU), jejunum (JEJ), ileum (ILE), caecum (CAE), sternal flexure (SF), pelvic flexure (PF), right dorsal colon (RDC), rectum (RE).

After slaughtering, the following intestinal tract compartments (described in Figure 1) were immediately identified and clamped before being opened for sample collection:

- duodenum
- jejunum
- ileum
- caecum
- sternal flexure
- pelvic flexure
- right dorsal colon
- rectum

### 2.3 DNA extraction and metataxonomic analysis

A total of 152 samples (8 intestinal tract compartments/horse for 19 horses) were subjected to DNA extraction and sequencing. DNA was isolated using the QIAamp Power Fecal Pro DNA Kit (QIAGEN, Milan, Italy) according to the manufacturer’s instructions. We added one microliter of RNase (50 mg/mL, Illumina Inc., San Diego, CA) to the DNA samples and incubated them for 1 h at 37°C to digest any RNA present. The DNA was normalized to 5 ng/μL and used as the template for the amplification of the V3-V4 region of the 16S rRNA gene (16S Amplicon PCR Forward Primer = 5’TCGTCGGCAGCGTCAGATGTGTATAAGAGACAGCCTACGGGNGGCWGCAG; 16S Amplicon PCR Reverse Primer = 5’GTCTCGTGGGCTCGGAGATGTGTATAAGAGACAGGACTACHVGGGTATCTAATCC) (28). The PCR products were purified and tagged with the Nextera index according to the 16S Metagenomic Sequencing Library Preparation from Illumina (Illumina, San Diego, USA). We sequenced the libraries on the Illumina MiSeq platform (Illumina Italy s.r.l., Milan, Italy) with V3 chemistry and generated 250 bp paired-end reads according to the manufacturer’s instructions.

### 2.4 Bioinformatics and statistical analysis

Each horse within each diet group was considered as experimental unit. After sequencing, we analyzed the raw reads using Quantitative Insights into Microbial Ecology (QIIME) 2 software (29). Following denoizing, performed by DADA2 (30), amplicon sequence variants (ASVs) were used for taxonomic assignment using the QIIME feature-classifier classify-consensus-vsearch (31) plugin against the greengenes database (v.13). We then used the QIIME-diversity script (29) to calculate the alpha and beta diversity parameters, which were then subjected to pairwise comparisons to assess for differences in the microbiota composition in the different intestinal tract compartments according to the diet used (HF vs. HS). In particular, we analyzed the indices of alpha diversity (Chao1, Shannon and the observed species richness) using Kruskal–Wallis tests, and we compared the beta diversities by pairwise PERMANOVA analysis based on the Bray-Curtis distance matrix using the QIIME2 script (29).

We imported the ASVs (Amplicon sequence variants), at the lowest taxonomic level (family or genus), into R to build a heatmap by applying the function “made4” (32). Moreover, we used pairwise Kruskal-Wallis tests to assess for differences in microbial taxa abundance according to diet (HF vs. HS). *p*-values were adjusted for multiple testing using the false discovery rate (FDR) method (*p*-value adjustment methods Benjamini-Hochberg). We considered a FDR < 0.05 as significant. The Picrust2 (Phylogenetic Investigation of Communities by Reconstruction of Unobserved States) software package (33) was then used to predict the potential functions of the microbiota. KEGG orthologs were collapsed at level 3 of hierarchy, and the table imported into the GAGE Bioconductor package (34) to identify the potential pathways either under-or overrepresented in the samples from the HF vs. HS dietary groups.

## 3 Results

At the end of the fattening period, we revealed no significant difference in the mean ± SD slaughter BW of horses fed the HF vs. the HS diet: 344.40 ± 2.91 kg vs. 347.67 ± 6.71 kg, respectively (15). No clinical signs of colic or gastric ulcerations were recorded during the study period but the horses belonging to the HS group produced unformed feces. Moreover, histopathological evaluation of the different intestinal tract compartments showed higher level of inflammation in the jejunum and pelvic flexure of horse fed the HS diet compared with those fed the HF diet (25).

### 3.1 Alpha and beta diversity

After sequencing and denoizing, a total of 565,643 raw reads were used for downstream analysis, corresponding to a sample coverage exceeding 99%. First, we compared the measures of alpha and beta diversity to investigate the effect of diet on microbiota diversity. Alpha diversity was measured by means of the Chao1 and Shannon indices and the observed species richness. As shown in Table 3, the Chao1 index was higher in the caecum of horses fed HF compared with HS (*p* = 0.01). The Shannon index was lower in the ileum of horses fed HF compared with HS (*p* = 0.04), and it was higher in the caecum (*p* = 0.04), pelvic flexure (*p* = 0.01), and right dorsal colon (*p* = 0.01) of horses fed the HF diet compared with the HS diet. Moreover, the observed species richness was significantly higher in the right dorsal colon of the horses belonging to the HF group compared with those belonging to the HS group (*p* < 0.01).

**Table 3:** Alpha diversity measures of microbiota composition in the selected intestinal tract compartments between horses fed the high-starch diet (HS) and the high-fiber diet (HF).

Intestinal tract compartment	Chao1		*p*-value	Shannon		*p*-value	Observed species richness		*p*-value
	HF	HS		HF	HS		HF	HS	
DU	338.77	287.02	0.60	5.62	6.15	0.26	217.67	189.11	0.60
JEJ	316.56	306.42	0.72	5.83	5.83	1.00	280.4	274.22	0.87
ILE	148.64	204.31	0.70	5.26	5.98	0.04*	139.3	188.13	0.66
CAE	611.60	543.48	0.01*	8.20	7.90	0.04*	574.20	491.78	0.07
SF	595.27	656.86	0.55	8.16	7.80	0.66	550.30	598.56	0.78
PF	689.05	619.08	0.32	8.47	7.93	0.01*	611.5	559.33	0.50
RDC	712.17	591.30	0.01*	8.47	7.86	0.01*	644.7	520.89	<0.01*
RE	551.05	703.04	0.24	8.13	8.29	0.84	568.33	710.1	0.50

*Statistical significance: *p* < 0.05. DU, duodenum; JEJ, jejunum; ILE, ileum; CAE, caecum; SF, sternal flexure; PF, pelvic flexure; RDC, right dorsal colon; RE, rectum.

**Figure 2:**
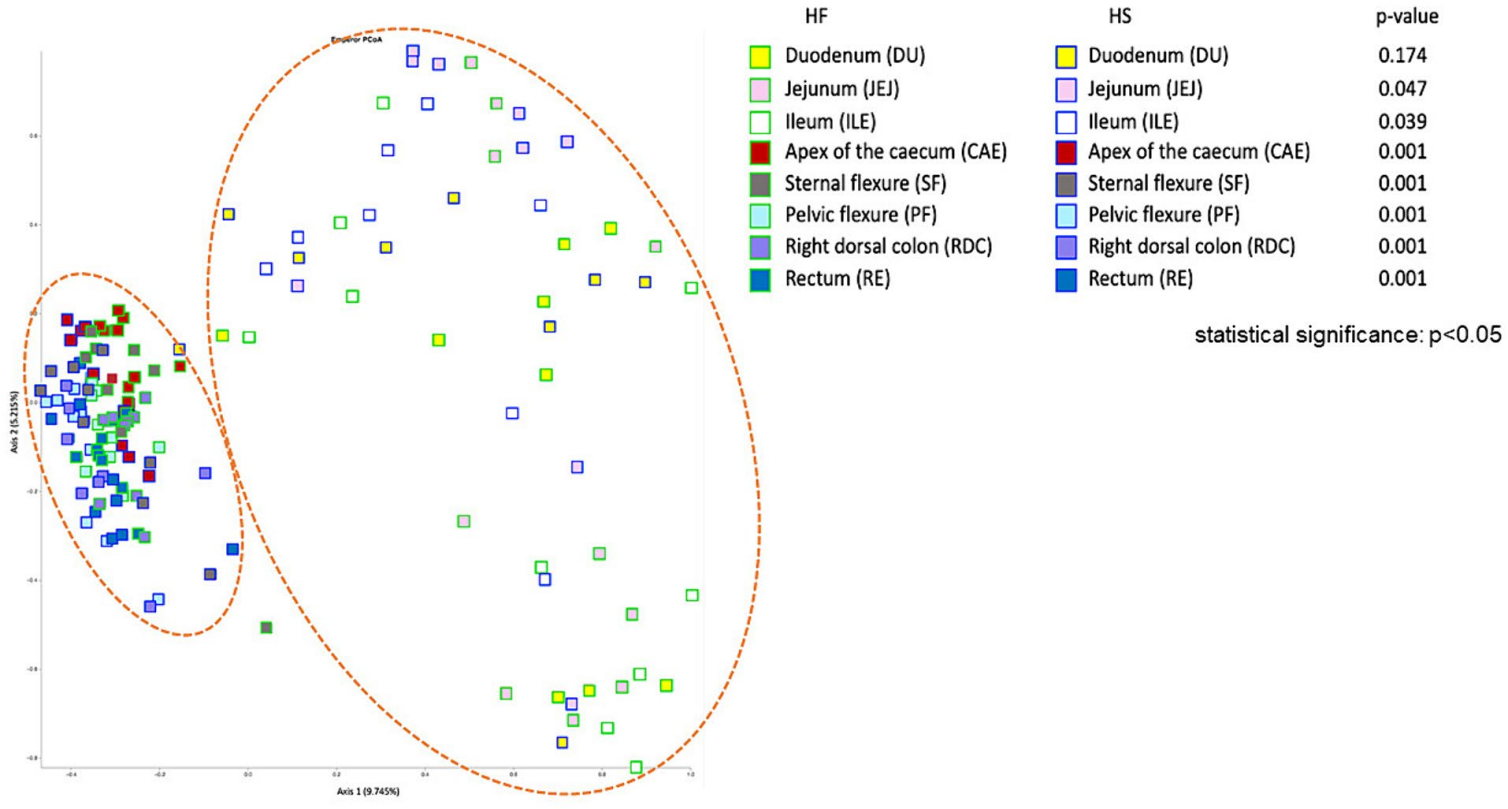
Principal Coordinate analysis (PCoA), based on the Bray Curtis distance, to study the effect of diet (high fiber, HF vs. high starch, HS) on the bacterial microbiota at different intestinal tract compartments of the horse.

To assess the effect of the two HF and HS diets on the microbiota composition at the different sampling locations, taking all the detected ASVs into account, we performed pairwise PERMANOVA analysis, based on the Bray Curtis distance matrix, to generate a measure of beta diversity. With the exception of the duodenum, the microbiota of each tract sampled differed significantly according to the feeding practice adopted (HF vs. HS, *p* < 0.05, Figure 2). These differences were demonstrated by the Principal Coordinate analysis based on the Bray Curtis distance matrix (PCoA, Figure 2). Moreover, the samples formed two distinct clusters, differentiating between the upper (DU, JEJ, ILE) and lower (CAE, SF, PF, RDC, RE) digestive tract of the horse.

### 3.2 Characterization of the bacterial microbiota at the phylum level

**Figure 3:**
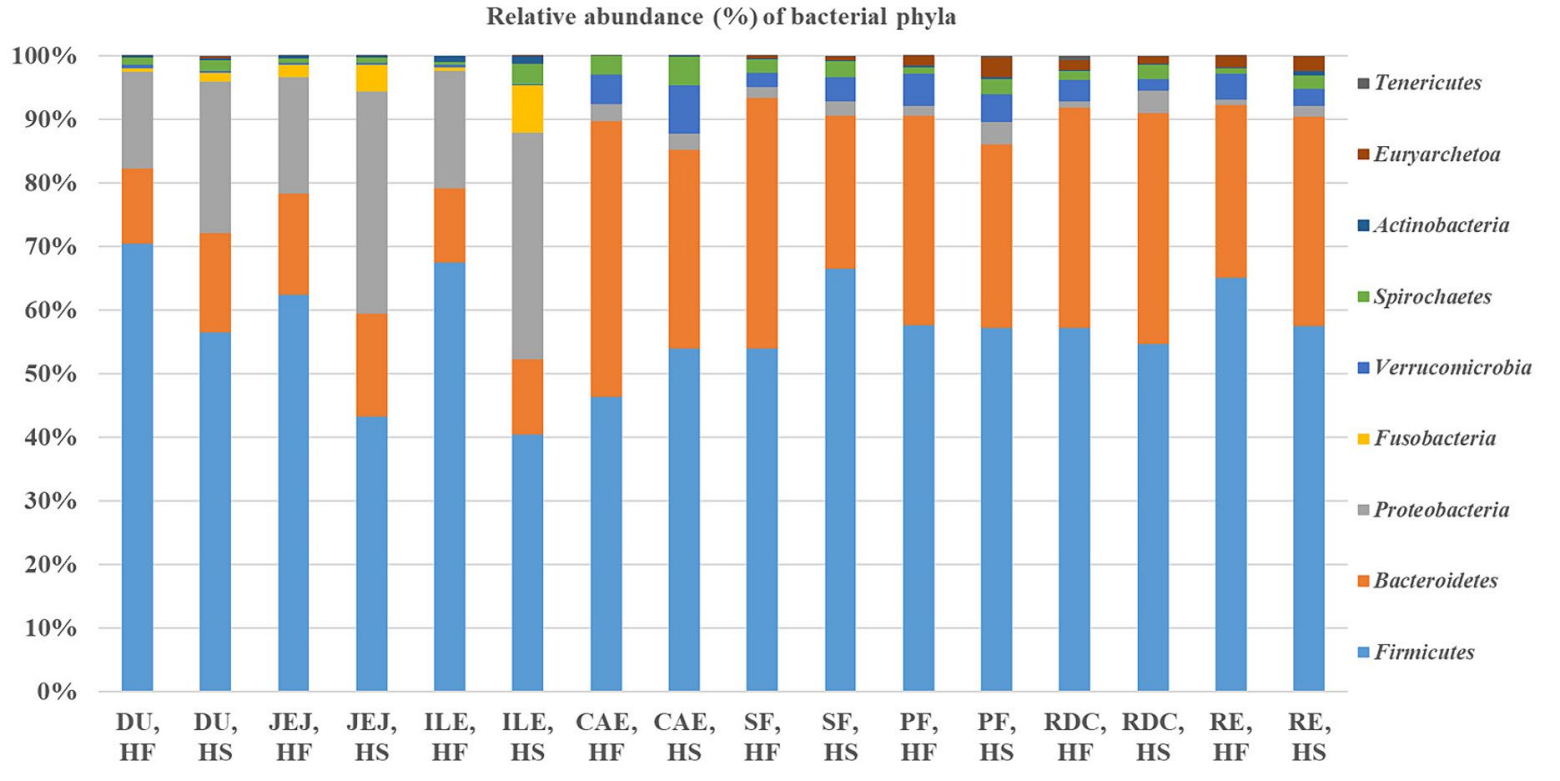
Relative frequency (%) of the bacterial phyla in the selected intestinal tract compartments (DU, duodenum; JEJ, jejunum; ILE, ileum; CAE, caecum; SF, sternal flexure; PF, pelvic flexure; RDC, right dorsal colon; RE, rectum) of the horses fed the high fiber diet (HF) and of the horses fed the high starch diet (HS).

The distribution and relative frequencies (%) of the phyla obtained by 16S rRNA gene sequencing are summarized in Figure 3. *Firmicutes* represented the dominant phylum in both HF and HS, outnumbering the *Bacteroidetes*, *Proteobacteria* and *Verrucomicrobia* phyla.

Considering the microbiota according to intestinal site, we found differences in the relative abundance frequencies of the different phyla. In particular, within the small intestine, the abundance of *Firmicutes* was greater in the ileum of HF compared to HS (67.4% HF vs. 40.4% HS; FDR < 0.05), whereas *Proteobacteria* were more prolific in the jejunum (18.3% HF vs. 35% HS) and ileum (18.4% HF vs. 35.6% HS) of HS compared with HF (FDR < 0.05). Within the hindgut, the frequency of *Firmicutes* in the sternal flexure of HS was higher compared with HF (53.9% HF vs. 66.5% HS; FDR < 0.05); whereas the frequency of the phylum *Bacteroidetes* was greater in horses receiving the HF diet in both the caecum (43.4% HF vs. 31.2% HS, FDR < 0.05) and the sternal flexure (39.4% HF vs. 24.1% HS, FDR < 0.05). Moreover, the relative frequency of *Spirochaetes* was higher in the rectum of horses fed the HS diet compared to the horses fed the HF diet (0.9% HF vs. 2.2% HS; FDR < 0.05).

### 3.3 Characterization of the bacterial microbiota at the genus level

**Figure 4:**
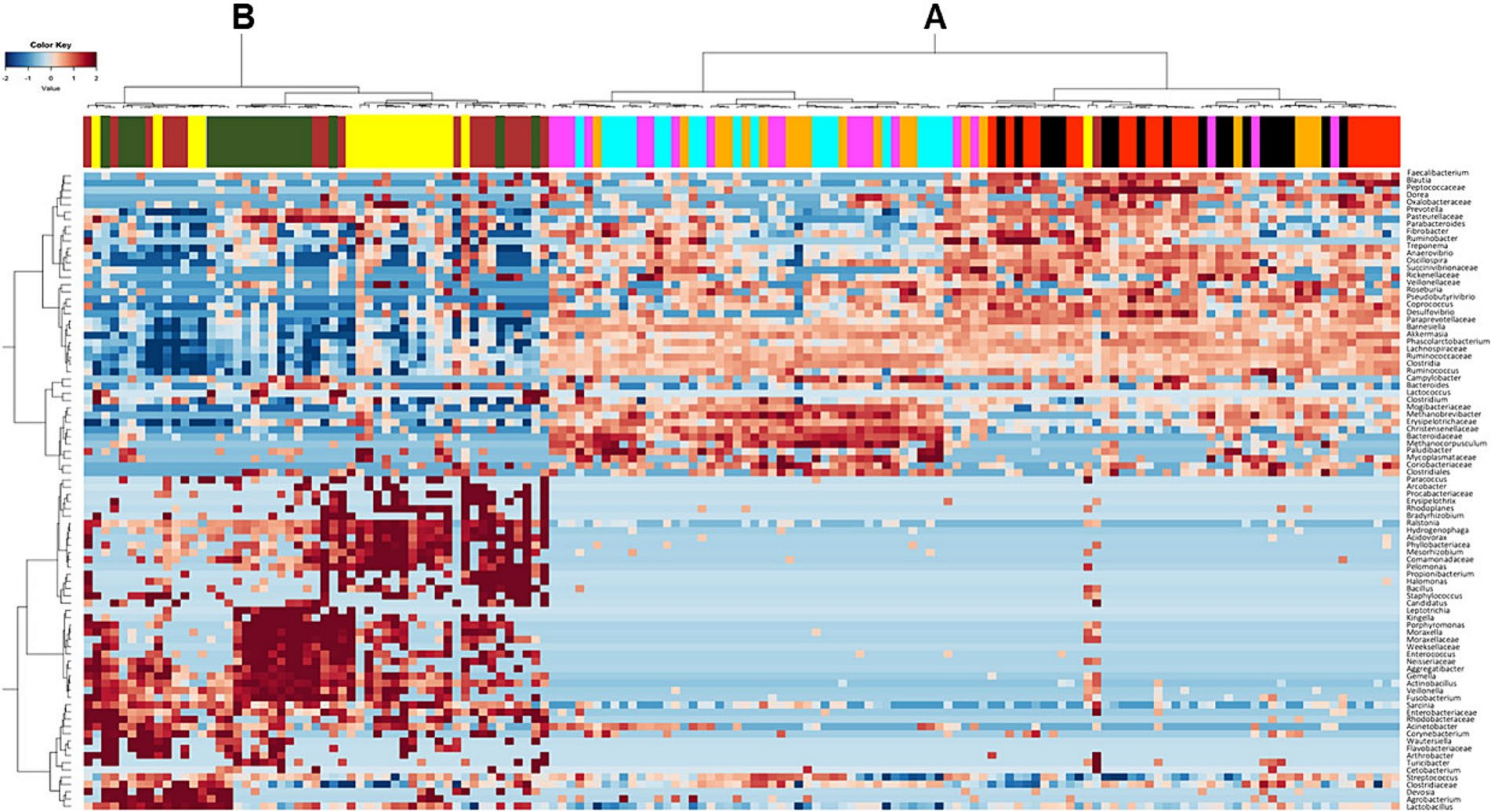
Heatmap showing the distribution of the Amplicon Sequence Variants (ASVs) detected in horses fed the high fiber diet (HF) and the high starch diet (HS) across the different intestinal tract compartments (duodenum = yellow, jejunum = dark green, ileum = brown, apex of the caecum = red, sternal flexure = black, pelvic flexure = pink, right dorsal colon = cyan, rectum = orange). The frequency of the ASVs is represented by color intensity, from dark blue (lowest frequency) through to dark red (highest frequency).

At lowest taxonomic level, cluster analysis of the most frequent ASVs confirmed the presence of distinct clusters (Figure 4). One cluster (A) comprised most of the samples collected from the hindgut (caecum, sternal flexure, pelvic flexure, right dorsal colon and rectum). Moreover, within the cluster A, a clear distinction was evident between the caecum and the sternal flexure, and between the pelvic flexure, right dorsal colon and rectum. The second cluster (B) comprised most of the samples collected from the duodenum, jejunum, and ileum.

**Figure 5:**
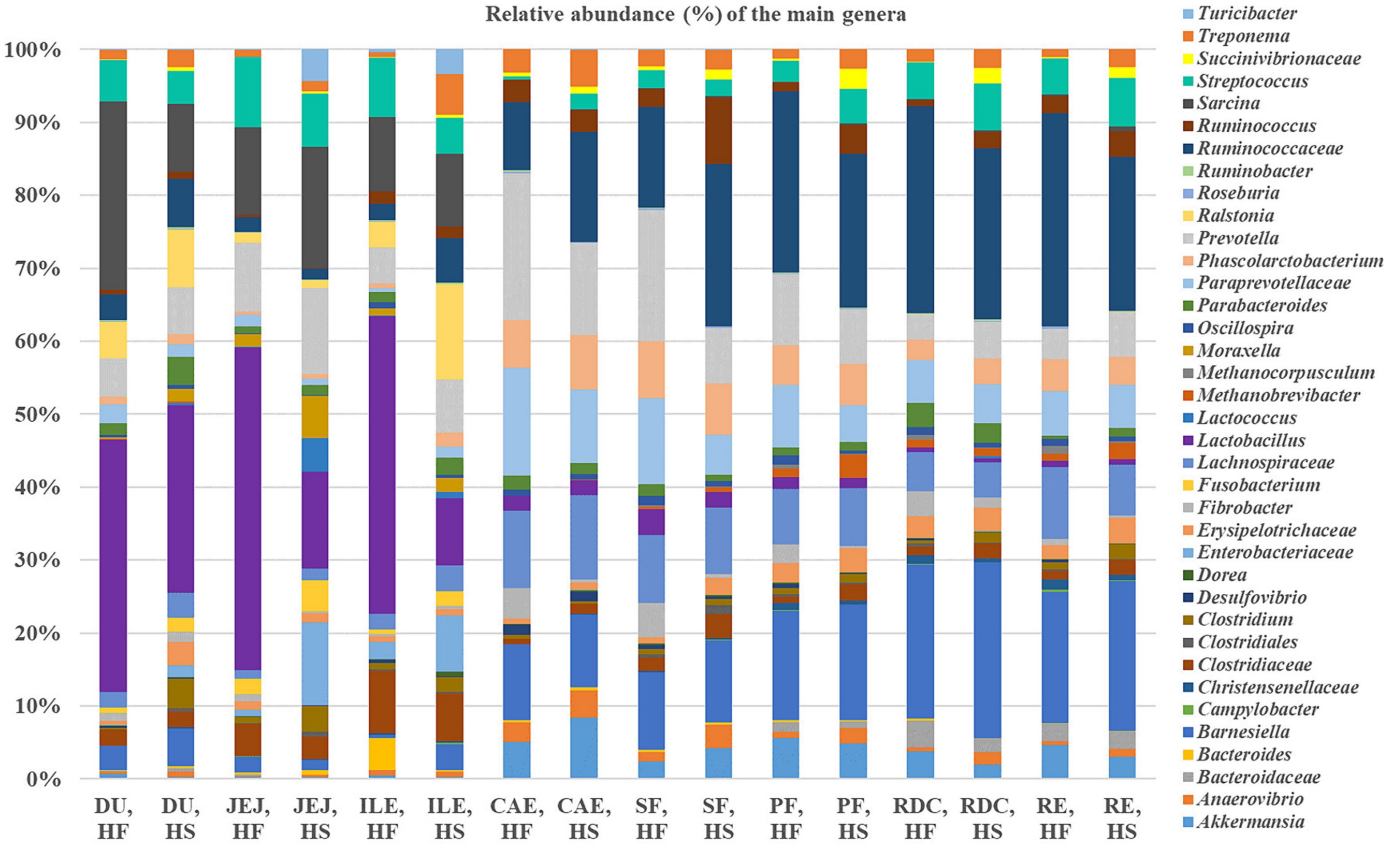
Relative frequencies (%) of the main genera across the different intestinal tract compartments (DU, duodenum; JEJ, jejunum; ILE, ileum; CAE, caecum; SF, sternal flexure; PF, pelvic flexure; RDC, right dorsal colon; RE, rectum) according to dietary group (high fiber, HF and high starch, HS).

The distribution of the main genera found along the different compartments of the intestinal tract according to the diet received is shown in Figure 5. The comparison of the relative frequency of ASVs in the duodenum between the two groups (HF vs. HS) showed greater levels of both *Clostridium* (0.1% HF vs. 2.9% HS; FDR < 0.05) and *Moraxella* (0.2% HF vs. 1.2% HS; FDR < 0.05) in horses fed HS.

In the jejunum, *Anaevibrio* (0.1% HF vs. 0.3% HS), *Lactococcus* (0% HF vs. 3% HS) and *Turicibacter* (2.9% HS vs. 0% HF) were all higher in horses fed the HS diet compared with the horses fed the HF diet (FDR < 0.05); whereas *Fibrobacter* (0.7% HF vs. 0.1% HS) and *Lactobacillus* (33.2% HF vs. 8.7% HS) were higher in the horses fed the HF diet compared to the HS diet (FDR < 0.05). A significant difference was found for *Enterobacteriaceae* which were more abundant in HS than HF (0.7% HF vs. 7.5% HS; FDR < 0.05).

By comparing the relative frequencies of the ASVs in the ileum, we found that *Phascolarctobacterium* was more abundant in horses fed HS than HF (0.5% HF vs. 1.1% HS; FDR < 0.05). The same was true for *Barnesiella* (0.4% HF vs. 2.1% HS; FDR < 0.05). Moreover, *Enterobacteriaceae* (0.7% in HF vs. 1.1% HS) and *Ralstonia* (2.6% HF vs. 7.8% HS) were higher in the ileum of the horses fed the HS diet compared to the horses fed the HF diet (FDR < 0.05).

The caecum was characterized by higher levels of *Fibrobacter* in HF compared with HS (3.8% HF vs. 0.3% HS; FDR < 0.05); whereas greater levels of *Streptococcus* were found in horses fed the HS diet (0.4% HF vs. 1.9% HS; FDR < 0.05).

Regarding the sternal flexure, *Fibrobacter* were greater in HF compared with HS (4.3% HF vs. 0.5% HS; FDR < 0.05), whereas *Anaevibrio* (1.2% HF vs. 2.9% HS) and *Erysipelotrichaceae* (0.8% HF vs. 2.1% HS) were significantly higher in HS (FDR < 0.05). *Paraprevotellaceae* (10.8% HF vs. 4.9% HS) and *Prevotella* (16.5% HF vs. 6.9% HS) were higher in HF compared with HS (FDR < 0.05). Moreover, *Methanobrevibacter* were more abundant in HS compared with HF (0.3% HF vs. 0.6% HS; FDR < 0.05) and *Fusobacterium* was only detected in horses fed HS (0% HF vs. 0.04% HS; FDR < 0.05).

The comparison of ASVs in the pelvic flexure showed *Ruminococcus* (1.2% HF vs. 3.6% HS), *Anaevibrio* (0.7% HF vs. 2% HS), *Sarcinia* (0% HF vs. 0.2% HS) and *Roseburia* (0.1% HF vs. 0.2% HS) to be greater in HS compared with HF (FDR < 0.05); whereas *Dorea* (0.2% HF vs. 0.1% HS) and *Oscillospira* (1.1% HF vs. 0.4% HS) were greater in horses fed the HF diet compared with HS (FDR < 0.05). *Succinivibrionaceae* were more abundant in HS compared with HF (0.3% HF vs. 2.5% HS; FDR < 0.05).

The right dorsal colon was characterized by greater levels of *Clostridium* (0.4% HF vs. 1.2% HS), and *Ruminococcus* (0.7% HF vs. 2% HS) in horses fed the HS diet compared with the horses fed the HF diet (FDR < 0.05). *Clostridiales* were more abundant in HF compared with HS (0.4% HF vs. 0.1% HS; FDR < 0.05). *Prevotella* was more abundant in HS (3% HF vs. 4.5% HS; FDR < 0.05), whereas *Bacteroides* were only detected in HF (0.3% HF vs. 0% HS; FDR < 0.05). Moreover, *Akkermansia* (3.3% HF vs. 1.8% HS) and *Methanocorpusculum* (0.1% HS vs. 0.6% HF) were present at greater levels in HF compared with HS (FDR < 0.05).

Finally, samples collected from the rectum of the horses fed the HF diet revealed higher relative abundances of bacteria belonging to *Lachnospiraceae* (8.7% HF vs. 6.3% HS; FDR < 0.05) and *Ruminococcaceae* (25.5% HF vs. 18.8% HS; FDR < 0.05). The results also revealed greater levels of *Treponema* (0.9% HF vs. 2.2% HS; FDR < 0.05) and *Succinivibrionaceae* in horses fed the HS diet compared with those fed HF (0.9% HF vs. 2.2% HS; FDR < 0.05).

Regarding functional differences in the microbiota of the two dietary groups, analysis revealed the HS diet to bring about an enrichment in genera involved in fermentative pathways, such as glycolysis/gluconeogenesis (ko00010), pyruvate metabolism, (ko00620), methane metabolism (ko00680) and propanoate metabolism (ko00640). Conversely, the HF diet brought about a reduction in potential microbiota functions related to fatty acid degradation (ko00071), nitrogen metabolism (ko00910) and sulfur metabolism (ko00920).

## 4 Discussion

The present study looked for variations in the gut microbiota along the length of the digestive tract of healthy horses according to two diets. We compared the effects of a high starch diet (HS), which exceeds the safe recommended level of 1 gram of starch/kg BW/meal (8), and a high fiber diet (HF), which satisfies the nutritional requirements of the horse.

The available scientific literature confirms the negative effects of HS diets on the gut health and welfare of horses (35), but a systematic assessment of the microbiota across the different compartments of the digestive tract is lacking (19). Microbiota composition is influenced by age, breed and the feed management system applied (23, 36). To avoid effects associated with breed or age in the present study, we used horses of the same age (14.3 ± 0.7 months) and breed (Bardigiano breed). They were not homogenous for sex; however, this is unlikely to have influenced the results since no sex-dependent differences in the gut microbiota were found in different gastrointestinal tract compartments of the horse analyzed using the NGS approach (18, 37). Moreover, all the horses of the present study were housed under the same environmental conditions, and the only difference was the diet.

Microbial populations were analyzed from alpha and beta diversity, bacterial populations present, and their potential function within the digestive tract of the horses.

A first important finding was that the horses fed the HF diet showed higher alpha diversity indices in the caecum, pelvic flexure and right dorsal colon compared with those fed the HS diet. This is relevant because high levels of bacterial diversity have been associated with greater stability of the intestinal microbiota and greater colonization resistance against potentially pathogenic bacteria (38, 39). However, the Shannon index was increased in the ileum of the horses fed the HS diet, whereas the alpha diversity indices of the other intestinal tract compartments were not different between the two diets. This could be explained by the individual variability or by the fact that the duration of the feeding trial (72 days) was too short to induce any significant changes in the other intestinal tract compartments. Moreover, the measures of beta diversity revealed significant compositional differences between the different intestinal tract compartments studied according to diet (HF vs. HS). This was not surprising since the diet is well-known to be the main factor affecting microbiota composition in the horse (1).

As already reported in the scientific literature, our findings support the division of the equine digestive tract into two distinct regions according to microbiota composition: the upper (duodenum, jejunum and ileum) and the lower (caecum, sternal flexure, pelvic flexure, right dorsal colon and rectum) digestive tract (see Figure 1) (18, 37). Interestingly, cluster analysis (Figure 4) revealed further differentiation within the hindgut: one cluster comprised the caecum and sternal flexure, and another was made up of the pelvic flexure, right dorsal colon and rectum; once again, confirming the findings of Costa and colleagues (18) who showed that that neighboring intestinal tract compartments are more similar to each other.

We show *Firmicutes* to be the primary bacterial phylum in all intestinal tract compartments, in accordance with the findings by other authors (18, 24, 40). *Bacteroidetes* was the second most abundant phylum (41, 42), and the third was *Proteobacteria* in the small intestine and *Verrucomicrobia* in the hindgut (19).

When we looked at the microbiota composition at genus/family level, we found interesting differences according to the diet adopted (HF vs. HS). The small intestine (jejunum and ileum) of horses fed the HS diet displayed a higher relative abundance of *Proteobacteria* compared with horses fed the HF diet as a result of a higher relative abundance of *Ralstonia* and *Enterobacteriaceae*. The increased presence of bacteria belonging to *Proteobacteria* is reported to be associated with inflammatory intestinal diseases in the horse (41). In particular, *Enterobacteriaceae* comprises some pathogenic bacteria, such as *Escherichia Coli* and *Salmonella* spp., which can lead to dysfunctions of the intestinal barrier, generating a condition of leaky gut and enteritis (1). Previous research published by our research team on the same horses fed the HS diet showed they were characterized by greater levels of inflammation of the intestinal wall in the jejunum (25) and by higher levels of intestinal permeability (15). Thus, we can speculate that these conditions were triggered by shifts in the microbiota composition associated with the consumption of a HS diet characterized by an overabundance of bacteria belonging to the *Proteobacteria*.

Progressing toward the more distal compartments of the digestive tract, the caecum and sternal flexure appeared similar in terms of their microbiota compositions, as shown in the heatmap (Figure 4). However, significant differences at the genus level revealed some positive effects of the HF diet related to an enhancement in metabolic pathways involved in fermentation processes with the production of short chain fatty acids (SCFAs). Specifically, the horses fed the HF diet showed higher frequencies of *Fibrobacter* in their caecal microbiota communities with respect to those fed the HS diet. Moreover, the abundances of *Fibrobacter*, *Prevotella* and *Paraprevotellaceae* within the sternal flexure were also greater. Dougal et al. (39) identified *Fibrobacteraceae* and *Prevotellaceae* as being two of the most abundant members of the core bacterial communities in the horse intestinal tract. Therefore, in our study the HS diet seems to be associated with a reduction in the overall size of the core microbiota within the caecum and sternal flexure. Such a reduction has been reported to increase the risk for digestive diseases and metabolic dysfunctions in the horse (43). Moreover, we found higher abundances of *Streptococcus* in the caecum and *Fusobacterium* in the sternal flexure of horses fed the HS diet, both of which are potential pathogenic bacteria associated with the presence of colitis or diarrhea (44). Indeed, the feces of horses fed the HS diet were less formed compared with those fed the HF diet (16).

Considering the distal intestinal tract compartments (pelvic flexure, right dorsal colon and rectum), *Succinivibrionaceae* were more abundant in the pelvic flexure and rectum of horses fed the HS diet. This is not surprising since these bacteria are amylolytic, and their greater prevalence is likely to be associated with the presence of undigested starch in the hindgut (26, 45). The samples collected from the pelvic flexure and right dorsal colon of both groups of horses showed compositional differences related to genera belonging to *Firmicutes* and *Bacteroidetes*. The majority of these taxa (*Clostridium*, *Clostridiales*, *Ruminococcus*, *Prevotella* and *Bacteroides*) are typical members of the physiological microbiota of the horse. However, it is interesting to note the positive effect that the HF diet had on the right dorsal colon. Samples collected from the right dorsal colon of horses fed the HF diet displayed a greater relative abundance of *Akkermansia*, a mucin-degrading genus that helps to maintain the integrity of the mucin layer of the intestinal barrier (19, 46). Finally, the fecal samples collected from the rectum of the horses fed the HF diet displayed an increase in the relative frequency of bacteria belonging to the *Lachnospiraceae* and *Ruminococcaceae* families, which form part of the intestinal core microbiota of the horse (39). Moreover, *Spirochaetes* (in particular *Treponema*), although at a lower abundance frequency than *Lachnospiraceae* and *Ruminococcacea*, were higher in the fecal samples of the horses fed the HS diet. This was an unexpected finding since these bacteria are involved in fiber degradation.

To the best of our knowledge, the present study is the first to evaluate the microbiota composition of all digestive tract compartments in horses homogenous for breed, age and previous management practices. A limitation of the present study was its small sample size. A second was the fact that the animals were not used as their own controls through comparison of their fecal microbiota. Studying the fecal microbiota before and after the feeding trial would have enabled us to discern whether limited changes of the microbiota differences in certain areas of the digestive tract between HF and HS groups were indeed attributable to the microbial composition of the ecological niches or a reflection of variability between horses. Nonetheless, the study provides evidence sustaining the view that each compartment of the intestinal tract behaves as an ecological niche, as first suggested by Costa and colleagues (18). Moreover, we demonstrated that insights into changes occurring at the lower taxonomic level along the length of the digestive tract (such as those caused by diet) cannot be gained by analyzing fecal samples collected from the rectum (which revealed a reduction of the overall size of the core bacterial community and an increase in amylolytic bacteria in the horses from the HS group). This finding has important practical implications for the design of studies aimed at investigating changes in the horse microbiota associated with certain variables, such as diet.

## 5 Conclusion

This is the first study to evaluate the effects of a high-fiber (HF) vs. a high-starch (HS) diet on the microbiota within the different intestinal tract compartments of healthy horses, homogenous for age and breed. The results confirm that each intestinal tract compartment of the horse’s digestive system represents a distinct ecological niche, and that diet significantly influences the microbiota composition. In particular, the overall size of the core community and the indices of alpha diversity were lower in horses fed the HS diet, which also showed higher levels of amylolytic bacteria. From the clinical perspective, our findings underscore the need to be cautious when drawing conclusions about the intestinal health of horses based on the analysis of the fecal microbiota. Fibrous diets should be promoted to prevent dysbiosis of the microbiota associated with high-starch diets.

## Data availability statement

The data presented in the study are deposited in the NCBI repository, accession number PRJNA1105226.

## Ethics statement

The animal studies were approved by Ethics Committee of the Department of Veterinary Sciences of the University of Turin (Italy) (Prot. N. 2202/2019). The studies were conducted in accordance with the local legislation and institutional requirements. Written informed consent was obtained from the owners for the participation of their animals in this study.

## Author contributions

FR: Conceptualization, Data curation, Investigation, Methodology, Writing – original draft. SC: Data curation, Methodology, Resources, Writing – review & editing. DB: Project administration, Resources, Supervision, Writing – review & editing. PS: Methodology, Resources, Supervision, Writing – review & editing. IF: Formal analysis, Methodology, Software, Writing – review & editing. LC: Data curation, Formal analysis, Methodology, Software, Writing – review & editing. MC: Data curation, Formal analysis, Methodology, Software, Writing – review & editing. RM: Methodology, Writing – review & editing. DC: Data curation, Formal analysis, Writing – review & editing. EV: Conceptualization, Project administration, Resources, Supervision, Writing – review & editing.
